# A long term global daily soil moisture dataset derived from AMSR-E and AMSR2 (2002–2019)

**DOI:** 10.1038/s41597-021-00925-8

**Published:** 2021-05-27

**Authors:** Panpan Yao, Hui Lu, Jiancheng Shi, Tianjie Zhao, Kun Yang, Michael H. Cosh, Daniel J. Short Gianotti, Dara Entekhabi

**Affiliations:** 1 Department of Earth System Science, Tsinghua University, Beijing, 100084 China; 2 State Key Laboratory of Remote Sensing Science, Aerospace Information Research Institute, Chinese Academy of Sciences, Beijing, 100101 China; 3 National Space Science Center, Chinese Academy of Sciences, Beijing, 100190 China; 4 Hydrology and Remote Sensing Laboratory (HRSL), United States Department of Agriculture-Agricultural Research Service (USDA-ARS), Beltsville, MD 20705 USA; 5 Parsons Laboratory, Department of Civil and Environmental Engineering, Massachusetts Institute of Technology, Cambridge, MA 02139 USA

**Keywords:** Hydrology, Hydrology

## Abstract

Long term surface soil moisture (SSM) data with stable and consistent quality are critical for global environment and climate change monitoring. L band radiometers onboard the recently launched Soil Moisture Active Passive (SMAP) Mission can provide the state-of-the-art accuracy SSM, while Advanced Microwave Scanning Radiometer for EOS (AMSR-E) and AMSR2 series provide long term observational records of multi-frequency radiometers (C, X, and K bands). This study transfers the merits of SMAP to AMSR-E/2, and develops a global daily SSM dataset (named as NNsm) with stable and consistent quality at a 36 km resolution (2002–2019). The NNsm can reproduce the SMAP SSM accurately, with a global Root Mean Square Error (RMSE) of 0.029 m^3^/m^3^. NNsm also compares well with *in situ* SSM observations, and outperforms AMSR-E/2 standard SSM products from JAXA and LPRM. This global observation-driven dataset spans nearly two decades at present, and is extendable through the ongoing AMSR2 and upcoming AMSR3 missions for long-term studies of climate extremes, trends, and decadal variability.

## Abstract (summary)

**Table:** 

Measurement(s)	wetness of soil • surface soil
Technology Type(s)	microwave spectroscopy • satellite imaging
Factor Type(s)	temporal resolution • spatial resolution
Sample Characteristic - Environment	planetary surface
Sample Characteristic - Location	global

Machine-accessible metadata file describing the reported data: 10.6084/m9.figshare.14533554

## Background & Summary

Surface soil moisture(SSM) is a key variable in water and energy exchange between land and atmosphere, which controls the partitioning of precipitation into runoff, evapotranspiration, and infiltration, as well as the partitioning of turbulent energy fluxes into latent and sensible heat^1–5^. As a Global Climate Observing System “Essential Climate Variable”^6,7^, long and spatio-temporally consistent SSM datasets are necessary for many applications including numerical weather prediction, disaster monitoring of drought and flood, agriculture yield assessment and water resource management, and also for numerical model and global climate change process studies.

Microwave remote sensing—particularly L-band radiometery—provides unique monitoring opportunity, with global coverage and high accuracy. Legacy and currently-operational satellites/sensors including SMAP and Soil Moisture and the Ocean Salinity mission (SMOS) working at L band (1.41 GHz), AMSR-E/AMSR2, ASCAT, FY-3, and TMI working at C band(6.9 GHz) or X band(10 GHz) *et al*., provide SSM products covering more than thirty years. Those products vary in accuracy and resolution as determined by the mission specifications, calibration, and the retrieval algorithms. The SMAP mission, launched by the National Aeronautics and Space Administration (NASA) in 2015^8^, provided observations of both Brightness Temperature (TB) and backscatter at L-band. SMAP has a dedicated subsystem to enable detection and mitigation of radio-frequency interference (RFI)^8^, to overcome L-band RFI problems encountered by SMOS^9,10^. Many validation and evaluation studies^11–13^ conducted at global and regional scales constrain errors in the SMAP SSM product to less than 0.04 m^3^/m^3^—useful for global monitoring—but longer time series are required for analyses of trends, decadal variability, and climatological studies. Meanwhile, the Japan Aerospace Exploration Agency’s (JAXA) AMSR2 (2012-present), combined with its predecessor the AMSR-E (2002–2011), have provided a set of TB covering 20 years with similar instrument attributes (frequencies, incident angles, etc.), orbit setting, TB calibration and processing. However, compared to the lower frequency SMAP SSM products, the currently available SSM products retrieved from ASMR-E/2 TB are more prone to errors and biases from atmospheric, vegetative, and soil interactions.

The usual method to establish a longer remote sensing dataset is to combine multiple satellite products. Owe *et al*.^14^ built a SSM dataset from 1978 to 2007 by applying one retrieval model (the land parameter retrieval model—LPRM) to the entire TB from available satellite microwave sensors working at C/X band. Liu *et al*.^15,16^ combined multiple soil moisture products covering 1978 to 2008, using a Cumulative Distribution Function (CDF) matching technique. This led to the Climate Change Initiative (CCI) program initiated by the European Space Agency (ESA), and its target for soil moisture is to produce a complete, consistent global SSM record based on active/passive products^17^. CCI SSM provides three datasets: an active dataset, a passive dataset, and a combined dataset. For the passive dataset, multiple satellites’ SSM products are retrieved using LPRM algorithm, and those products are rescaled by CDF matching. In the study of SSM fusion funded by ESA, De Jeu *et al*.^9,18–20^ developed three fusion approaches to retrieve SSM dataset using SMOS and AMSR-E. Yao *et al*.^21^ developed a dataset using SMOS and AMSR-E/AMSR2 using neural networks.

Of these long term satellite SSM datasets, only the CCI dataset is public and accessible. And while the CCI dataset is of sufficient length for climatological studies, a) the dependence on numerous sensor designs, frequencies, and retrieval algorithms, and b) the SSM CDF-matching approach preclude the assessment of climatological metrics such as trends and assessment of inter-annual to decadal scale variability.

This research extends some of the benefits of the SMAP SSM product to the longer AMSR-E/AMSR2 TB dataset using an ANN method to extract as many characteristics of the more robust L-band data from the higher frequency instruments. This approach allows us to transfer the merits of SMAP products back to AMSR2/AMSR-E TB and future AMSR3 TB. Currently, we have created a long term daily SSM product (named as ‘NNsm’) at 36 km resolution from 2002 to 2019. Validation against *in situ* data from the USDA, the Tibetan Plateau, OZNET, REMEDHUS and AMMA sites demonstrate that the NNsm matches well with SMAP and *in situ* SSM, with similar accuracy to SMAP (~0.04 m^3^/m^3^). Our intent is for this dataset to provide opportunities for longer-term study of the water cycle into the L-band era.

## Methods

The satellite brightness temperature (TB) data we use are: (1) AMSR-E TB in slow rotation mode (L1S), (2) AMSR-E and AMSR2 Level 3 standard TB. The AMSR-E/2 Level 3 daily TB at 0.25 degree resolution were obtained from the online dissemination service Globe Portal System (G-Portal, https://gportal.jaxa.jp/gpr/) of JAXA. The SMAP Level 3 passive daily SSM product (SMAPL3sm) was obtained from NASA National Snow and Ice Data Center Distributed Active Archive Center (NSIDC DAAC, https://nsidc.org/data/SPL3SMP/versions/6), with a 36 km resolution at cylindrical Equal Area Scalable Earth Grid, Version 2.0 (EASE-Grid 2.0), with size of 406 rows × 964 columns. Table 1 summarizes the data used, their spatial resolutions, and provenance.

**Table 1:** Details of data used in the study.

Data	Source	Time Period	Spatio-temporal resolution
AMSR-E L1S TB	https://gportal.jaxa.jp/gpr/	Callibration:2012–2015	Swath,
AMSR2 TB	https://gportal.jaxa.jp/gpr/	Callibration:2012–2015	0.25°, Daily
SMAP soil moisture	https://nsidc.org/data/SPL3SMP	Training: 2015–2017	36 km, Daily
AMSR2 TB	https://gportal.jaxa.jp/gpr/	Training: 2015–2017	0.25°, Daily
AMSR-E TB	https://gportal.jaxa.jp/gpr/	Simulation: 2002–2011	0.25°, Daily
AMSR2 TB	https://gportal.jaxa.jp/gpr/	Simulation: 2012–2019	0.25°, Daily

**Fig. 1:**
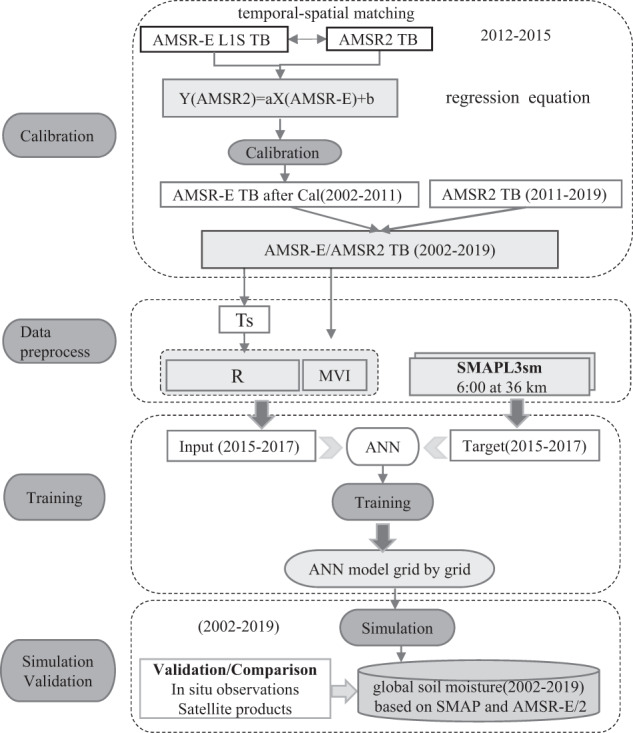
Data Processing for the NNsm dataset.

Our research approach adopt the soil moisture retrieval algorithm developed by Yao *et al*.^21^ using artificial neural networks (ANN). The approach is divided into three components: calibration, training, and simulation/validation, as shown in the Fig. 1. An ANN is a nonlinear mathematical computing system which is capable of representing arbitrarily complex nonlinear processes that relate the inputs and outputs of any system^22^. The structure of an ANN model includes an input layer, a hidden layer, and an output layer. We use MATLAB to implement the ANN training and simulation.

### AMSR-E/AMSR2 data inter-calibration

Firstly, to ensure consistency of data from two sensors onboard different satellites, the TB of AMSR-E are calibrated to the TB of AMSR2 for each grid cell.

The common approaches for inter-calibration^23^ are inappropriate for AMSR-E and AMSR2 data due to a temporal gap of 9 months between them. Previous study on inter-calibration for AMSR-E and AMSR2 are based on the double difference method(DD), taking a third sensor as an intermediate reference, such as the microwave radiometer imager (MWRI) onboard the FengYun-3B (FY3B) satellite^24–26^. AMSR-E operations were halted due to rotational issues in 2011, and then restarted at a slow rotation rate from 2012 to 2015 (L1S TB), which is useful for users who cross-calibrate AMSR-E with other radiometers. Hu *et al*.^27^ conducted direct global calibration for AMSR-E and AMSR2 using AMSR-E L1S TB data.

The AMSR-E L1S data has 3 years of overlapping observations with AMSR2, based on which we developed a linear regression model for each grid cell to calibrate the AMSR-E TB to AMSR2 TB, for each frequency and each polarization:

1 $$T{B}_{amsr2}=a\ast T{B}_{amsre}+b$$

Inputting the AMSR-E TB (2002–2011) into Eq. (1) yields the calibrated AMSR-E TB. Combined with AMSR2 TB (2012–2019), we get harmonized TB time series for AMSR-E and AMSR2 from 2002 to 2019.

#### Pre-process of AMSR-E L1S TB

**Fig. 2:**
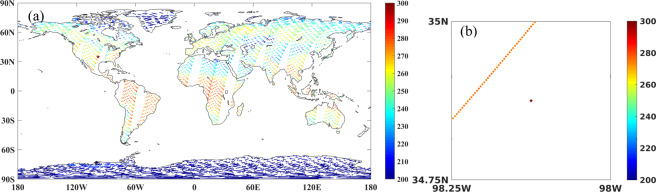
(**a**) Global daily map of AMSR-E L1S terrestrial TB (K) on April 11, 2015 (**b**) overlapping L1S TB (K) footprints for one overpass in one 0.25 degree grid cell covering Little Washita (LW) on April 11, 2015.

The global distribution of the slow-rotation L1S terrestrial data is shown for one day in Fig. 2(a). While the total observed area is sparse for any day, locally there are many observations with overlapping footprints. Figure 2(b) displays the centers of those overlapping footprints in one 0.25 degree grid cell at Little Washita. First, we resampled those observed points to 0.25 degree.

#### Inter-calibration over each grid cell

**Fig. 3:**
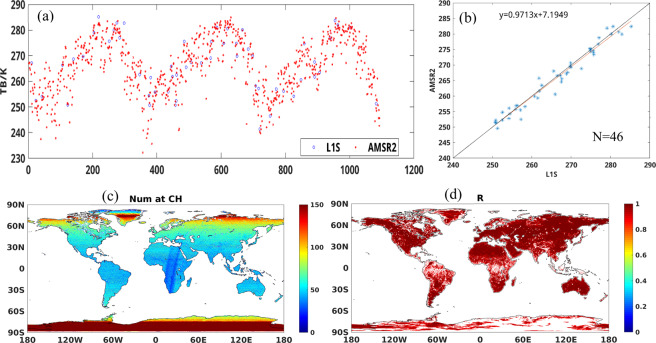
Inter-calibration reelation for the Little Washita Experimental Watershed: (**a**) time series for AMSR-E L1S TB and AMSR2 TB from Dec. 2012 to Dec. 2015, (**b**) scatterplot of two TB set together with the regression equation, (**c**) the global distribution of the number of matched-pair for C band and H-pol, (**d**) the global distribution of regression correlation coefficient R for AMSR-E L1S TB and AMSR2 TB inter-calibration from Dec. 2012 to Dec. 2015.

We carry out the inter-calibration over each grid cell. We take the grid cell of the Little Washita Experimental Watershed in southwestern Oklahoma, USA (LW) as an example. In Fig. 3(a), the blue circles are TB of L1S and the red dots are TB of AMSR2. Figure 3(b) shows the calibration equation for H polarization at C band for the grid cell. In Fig. 3(a), the blue circles are TB of L1S and the red dots are TB of AMSR2. We obtain the linear relationship using the matched-pair data (N = 46), with correction slope a = 0.9713, and regression R = 0.9820.

The number of matched-paird at C band for H-polarization is shown globally in Fig. 3(c).The number of matched-pairs per grid cell is greater than 40 at mid-latitudes, which ensures sufficient statistical power to determine the regression relationship. The regression R for each grid cell is shown in Fig. 3(d). Here we only display the grids where R > 0.85; 99.92% of locations show statistically significant inter-calibration relationships (P = 0.05).

#### General inter-calibration equations

In the equatorial zone and around the land-sea interface we find some pixels with regression R less than 0.85. We deem this calibration approach for these grid cells to be uncertain. Those areas are shown in white in Fig. 3(d). To still provide an estimated cross-sensor inter-calibration for these grid cells with fewer matched-pairs, we use a general inter-calibration relation for each frequency and each polarization estimated using all global matched-pairs for the 2012–2015 period. The regression coefficients for these general relationships are shown in Table 2. Users investigating hydroclimate regime change or decadal shifts in mean state at the single-pixel level, however, should be aware of the methodological difference for these locations. A mask marking pixels using the global inter-calibration model is given with the dataset file.

**Table 2:** Global inter-calibration parameters for Eq. 1. Used for locations with unstable local inter-calibration. Location mask given in file Inter_cal_mask.nc.

Frequency(GHz)	H-pol		V-pol	
	a	b	a	b
6.9	0.9831	4.7708	0.9848	3.7934
10.65	0.9979	2.6223	0.9932	4.0322
18.7	0.9825	4.1716	0.9689	8.4616
23.8	0.9929	2.9569	0.9978	2.1383
36.5	0.9987	2.0096	1.0044	0.8706

### ANN Training

In the ANN training period from March 2015 to December 2017, the input layer is comprised of reflectivity (R) and the microwave vegetation index (MVI)^28^ derived from AMSR2 TB; the output layer (training target) is the SMAPL3sm^21^. Ten neurons are used in our net and the training function is the default ‘trainlm’ function.

#### Data pre-processing

The descending SMAPL3sm data (at 06:00 local time) are selected in this study, corresponding to the AMSR-E/2 descending nighttime TB data (at 01:30 local time). The AMSR-E/2 Level 3 daily descending TB data (01:30) at 0.25 degree resolution are selected. And those AMSR2 TB are resampled to the 36 km EASE-Grid 2.0 using linear interpolation. The R is derived from estimated surface temperatures (Ts)^29^ and TB, and the MVI is derived from AMSR-E/2 TB at C band and X band:

2 $${{\rm{R}}}_{f}^{p}=1-\frac{T{B}_{f}^{p}}{{T}_{s}}$$

3 $${T}_{s}=1.11\times T{B}_{v}^{36.5}-15.2$$

4 $${\rm{MVI}}\left({{\rm{f}}}_{1},{{\rm{f}}}_{2}\right)=\frac{{{\rm{TB}}}_{v}\left({{\rm{f}}}_{2}\right)-{{\rm{TB}}}_{h}\left({{\rm{f}}}_{2}\right)}{{{\rm{TB}}}_{v}\left({{\rm{f}}}_{1}\right)-{{\rm{TB}}}_{h}\left({{\rm{f}}}_{1}\right)}$$

where f is the frequency, p is the polarization, v is vertical polarization and h is horizontal polarization.

#### ANN Training for each grid cell

To determine the local relationships between AMSR2-derived R/MVI and SMAPL3sm, we trained ANNs for every EASE-Grid 2.0 grid cell. Our ANN network was designed to minimize Mean Squared Error (MSE) of SMAPL3sm, using a random internal assignment of data into training/validation/testing categories (these assignments are distinct from our external data division and were applied only to our 2015–2017 testing data), with Levenberg-Marquardt (L-M) optimization used for back propagation.

During the training period from 2015 to 2017, the target SMAPL3sm and the input (R and MVI) were matched grid cell by grid cell. We removed those cells with less than 50 matching pairs (N) of AMSR2-SMAP observations. We set the Ts derived from the AMSR2 TB as a criterion of the freeze/thaw, and removed the data for frozen states. The global ANN were trained grid cell by grid cell with matching data.

### NNsm simulation for each grid cell

In the simulation period from 2002 to 2019, the input R/MVI were derived from consistent AMSR-E/2 TB, as described in data pre-processing. Over frozen soil, soil moisture is not retrieved, thus we did not simulate SSM for grid cells when Ts was lower than 273.15 K. We do not mask values based on surface water or vegetation water content, and highlight that the target (SMAP) values are less reliable for high values of each, meaning that these results could be screened with the same methods as used for the SMAP data itself. With the pre-processed input and the global ANNs model for each grid cell trained in the previous step, we derived the daily soil moisture from 2002 to 2019 on each grid.

## Data Records

The data records^30^ contain global daily soil moisture data with a spatial resolution of 36 km, in m^3^/m^3^, from June 2002 to December 2019. These data are stored in NetCDF format with one file per day, defined by two dimensions (lat, lon, respectively representing latitude and longitude) and a variable soil moisture (soil_moisture). The file name is “yyyyddd.nc”, where “yyyy” stands for year and “ddd” stands for Julian date. For example, “2003001.nc” contains the global soil moisture distribution on the first day of 2003.

Naming convention:

yyyyddd.nc

Variable: soil_moisture=volumetric soil moisture [m^3^/m^3^]

## Technical Validation

### Training and simulation performance

To confirm the reliability of the algorithm, we first present the performance of the ANN model during the training period from 2015 to 2017.

**Fig. 4:**
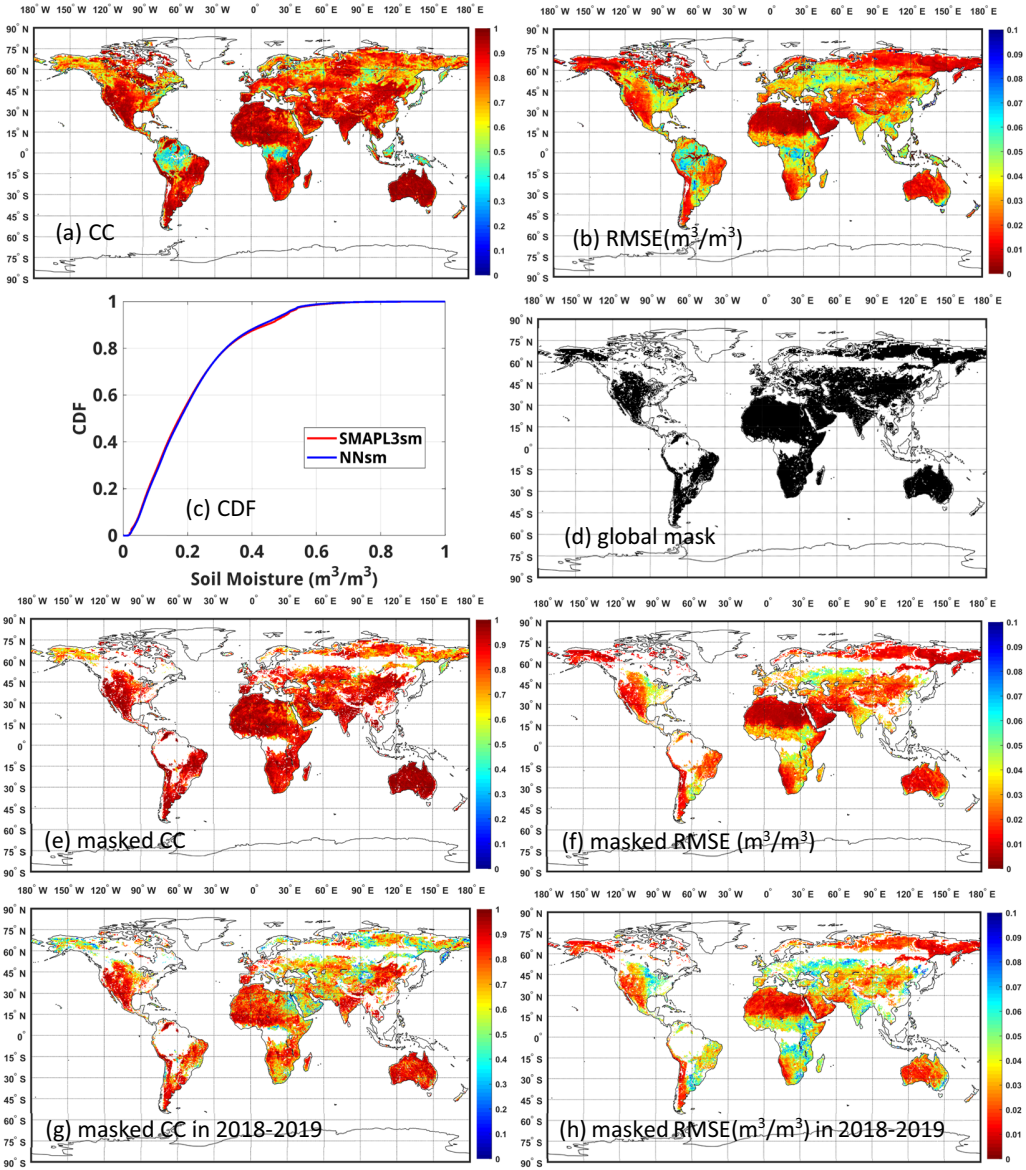
Global map of statistical comparison between NNsm and SMAPL3sm over training period (2015–2017): (**a**) Correlation coefficient, (**b**) Root mean squared error (m^3^/m^3^), (**c**) Cumulative distribution functions of all of the daily data for the NNsm and SMAPL3sm, (**d**) Global mask for regions with high vegetation water content, surface water, urban areas, and permanent ice/snow, (**e**) Masked correlation coefficients, (**f**) Masked RMSE, (**g**) Masked correlation coefficients (2018–2019), (**f**) Masked RMSE (2018–2019).

We evaluated the output of the trained ANN model, NNsm, against the target SMAPL3sm over the training period by analyzing the correlation coefficients and root-mean squared errors between the NNsm and the SMAPL3sm for each grid cell. The accuracy of predicted NNsm against the SMAPL3sm are shown in Fig. 4.

The trained NNsm values generally correlate well with the reference SMAPL3sm values over most of the globe, with a global mean of CC = 0.80, and a global mean of RMSE = 0.029 m^3^/m^3^. Globally, the CC values are high in regions with significant soil moisture dynamics. The RMSE values scale roughly with the local dynamic range of soil moisture and remain relatively small as a fraction of mean SSM values. Highly vegetated areas, including the Amazon and Congo rainforests, have correlations on the order of 0.5, presumably showing the AMSR-E/2 sensors’ diminished canopy penetration, as well as larger uncertainty in retrievals at all microwave frequencies for grid cells with high vegetation water content. Areas with commonly frozen ground also show reduced correlations and larger RMSEs. From the Cumulative Distribution Functions (CDF) of all of the daily data for the NNsm and SMAPL3sm in Fig. 4(c), we can see that the two CDF curves are very similar, with NNsm appearing to be a little drier than SMAPL3sm in the wettest conditions.

Figure 4(d) shows a global mask in black, where SMAP is expected to have errors less than 0.04 m^3^/m^3^. Surfaces with permanent ice and snow, urban areas, wetlands, and vegetated areas with vegetation water content >5 kg/m^2^ are excluded. The masked results of Fig. 4(a,b), removing the white areas from Fig. 4(d), are shown in Fig. 4(e,f). The spatial mean CC for Fig. 4(e) is 0.87 and the spatial mean RMSE for Fig. 4(f) is 0.022 m^3^/m^3^ as shown in Table 3.

**Table 3:** Global statistical comparison between NNsm and SMAPL3sm over training period (2015–2017) and simulation period(2018–2019). Values are the spatial means of individual pixel values.

	CC	RMSE(m^3^/m^3^)	Bias
Global training	0.80	0.029	0.0017
Global training masked	0.87	0.022	−0.0001
Global simulation masked	0.73	0.033	−0.0014

Data in 2018–2019 was used to validate the performance of trained NNsm. As shown in Fig. 4(g,h). The correlation coefficients and RMSE between trained NNsm and SMAPL3sm has similar distribution pattern with that of 2015–2017, but there’s a slight decrease in accuracy than that of training period, with a spatial mean of masked CC = 0.73, and a spatial mean of masked RMSE = 0.033 m^3^/m^3^.

### Validation using *in situ* observation

The performance of the simulated long term NNsm was validated against *in situ* soil moisture observations for the period from 2002 to 2019. We use *in situ* soil moisture observations from 14 representative validation sites, including (a) 7 United States Department of Agriculture (USDA) watershed sites (Walnut Gulch, Little Washita, Fort Cobb, Little River, Saint Joseph’s, South Fork, and Reynolds Creek), (b) 2 Tibetan Plateau sites (Pali and Naqu), (c) 2 Australian Moisture Monitoring Network (OzNet) sites (Yanco and Kyeamba), (d) the REMEDHUS Network sites, and (e) 2 African Monsoon Multidisciplinary Analysis (AMMA) sites (Benin and Niger) as shown in Fig. 3 and Table 4. Data of OzNet, REMEDHUS and AMMA sites are provided by International Soil Moisture Network (ISMN) (https://ismn.geo.tuwien.ac.at/) website^31–34^.

**Table 4:** List of validation sites. Time series for bold-face sites are shown in Fig. 6.

	Location	Site Name	Climate regime^a^	IGBP^b^ Land cover
1	USDA (North America)	Walnut Gulch	Arid	Shrub open rangeland
2		**Little Washita**	Temperate	Grasslands/Cropland mosaic
3		Fort Cobb	Temperate	Croplands
4		Little River	Temperate	Cropland/natural mosaic
5		Saint Joseph’s	Cold	Croplands
6		South Fork	Cold	Croplands
7		Reynolds Creek	Arid	Grasslands
8	Tibetan Plateau (Asia)	Pali	Arid	Barren/sparse
9		**Naqu**	Polar	Grasslands
10	OZNET (Australia)	**Yanco**	Semi-arid	Croplands/Grasslands
11		Kyeamba	Temperate	Croplands
12	REMEDHUS (Europe)	**REMEDHUS**	Temperate	Croplands
13	AMMA (Africa)	**Benin**	Arid	Savannas
14		Niger	Arid	Grasslands

^a^Koeppen-Geiger climate classification^38^.

^b^International Geosphere-Biosphere Program.

**Fig. 5:**
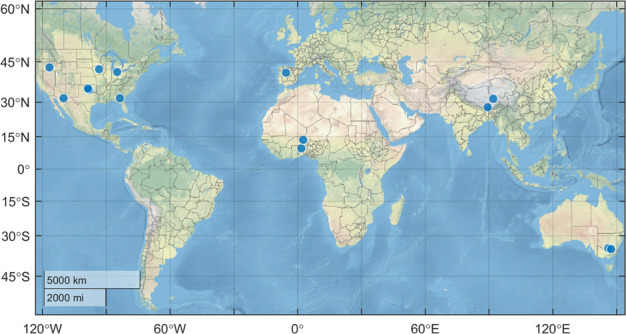
Location of *in situ* soil moisture validation sites.

These ground-based sites are major validation points for satellite soil moisture products, covering a wide variety of topography, land cover types and soil types around the world. These sites, which include dozens of instrumented stations each, are designed to provide reliable and representative soil moisture values for comparison against spatially-aggregated satellite footprints. Fig. 5 shows the location of these sites.

**Fig. 6:**
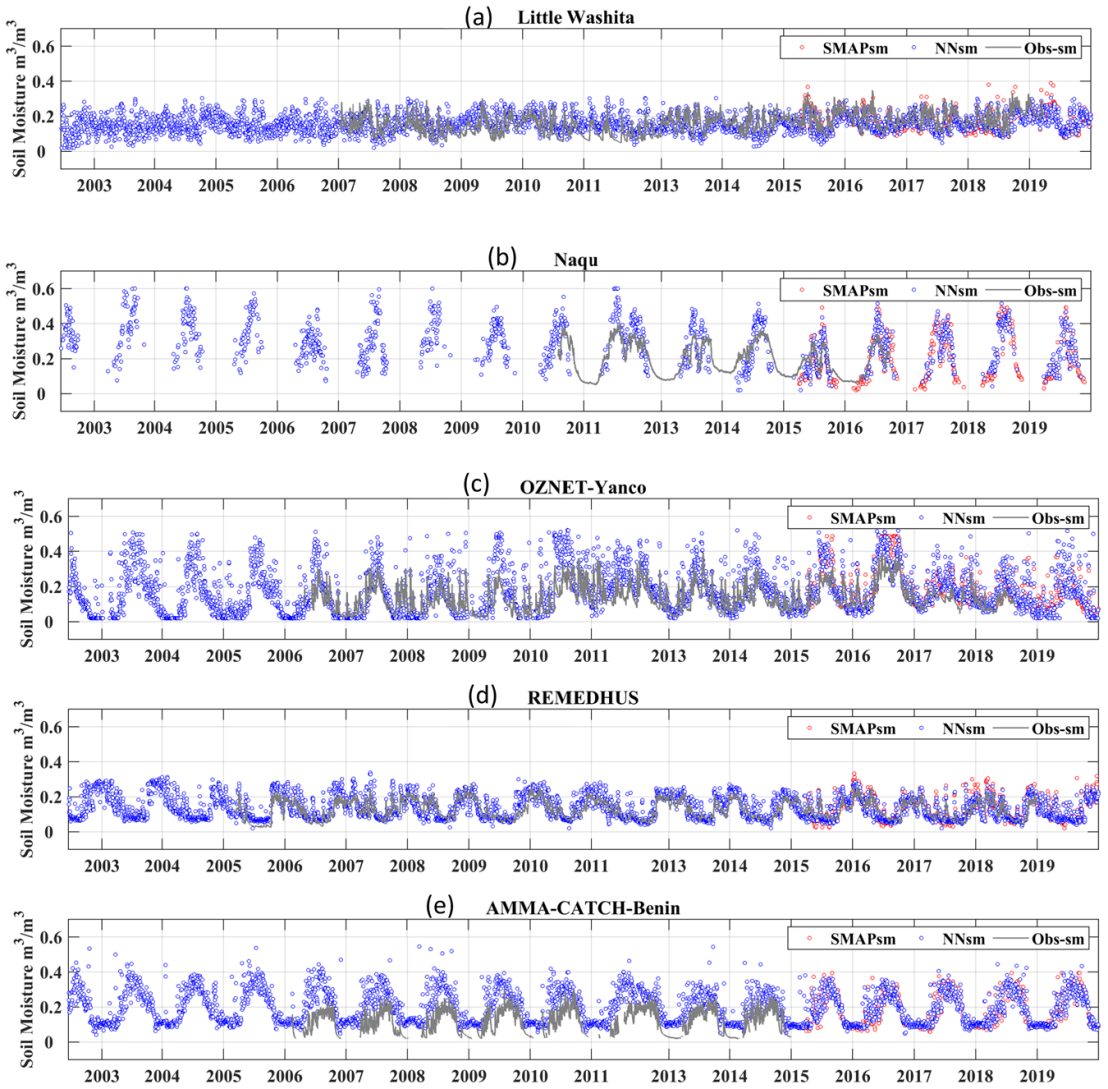
Time series of NNsm (blue dots), SMAPL3sm (red dots), and *in situ* soil moisture (Obs-sm in grey lines) for 2002–2019. *In situ* sites shown: (**a**) USDA-Little Washita, (**b**) Tibet-Naqu, (**c**) OzNet-Yanco, (**d**) REMEDHUS, and (**e**) AMMA-CATCH-Benin sites.

The performance of the NNsm over the ground sites are shown in time series in Fig. 6 and in a statistical matrix in Table 5. For demonstration, we show time series for one site in every continent. The magnitude and variability of NNsm (blue dots) are consistent with those of SMAPsm (red dots) and *in situ* SM (Obs-sm, grey line), and NNsm captures both the daily and seasonal dynamics of *in situ* SM. The ANN performs stably for both the training period (2015–2017) and the broader simulation period (2002–2014 and2018–2019), which can be seen both from the time series in Fig. 6 and the statistics in Tables 5 and 6 (2002–2019,in next section). Since the training target is SMAPL3sm, the performance of NNsm relative to *in situ* soil moisture (Obs-sm) is similar to SMAPsm as shown over most sites, and can do no better than SMAP in conditions where satellite and *in situ* soil moisture diverge.

**Table 5:** Statistical comparisons of NNsm and SMAPL3sm with the *in situ* soil moisture for training period (2015–2017) and simulation period(2018–2019).

2015–2017	NNsm vs. *in situ*				SMAPsm vs. *in situ*			
	CC	RMSE	Bias	ubRMSE	CC	RMSE	Bias	ubRMSE
1. Walnut Gulch	0.58	0.026	0.001	0.026	0.81	0.025	0.003	0.024
2. Little Washita	0.77	0.037	−0.017	0.033	0.88	0.030	−0.016	0.026
3. Fort Cobb	0.73	0.050	−0.029	0.040	0.86	0.043	−0.027	0.033
4. Little River	0.77	0.082	0.075	0.034	0.86	0.085	0.079	0.031
5. Saint Joseph’s	0.66	0.079	0.068	0.040	0.70	0.087	0.073	0.048
6. South Fork	0.50	0.041	0.011	0.040	0.59	0.064	0.017	0.062
7. Reynolds Creek	0.66	0.057	−0.022	0.053	0.72	0.053	−0.020	0.050
8. Pali	0.55	0.081	−0.074	0.032	0.75	0.075	−0.070	0.025
9. Naqu	0.80	0.069	−0.008	0.068	0.86	0.071	−0.008	0.070
10. Yanco	0.78	0.089	0.050	0.074	0.87	0.088	0.051	0.072
11. Kyeamba	0.74	0.103	0.068	0.077	0.85	0.098	0.053	0.083
12. REMEDHUS	0.82	0.033	0.002	0.033	0.81	0.039	0.004	0.038
13. Benin	—	—	—	—	—	—	—	—
14. Niger	—	—	—	—	—	—	—	—
**Average**	**0.70**	**0.062**	**0.010**	**0.046**	**0.80**	**0.063**	**0.012**	**0.047**
**2018**–**2019**	**NNsm vs**. ***in situ***				**SMAPsm vs**. ***in situ***			
	**CC**	**RMSE**	**Bias**	**ubRMSE**	**CC**	**RMSE**	**Bias**	**ubRMSE**
1. Walnut Gulch	0.78	0.028	0.008	0.027	0.83	0.029	0.007	0.029
2. Little Washita	0.74	0.039	−0.010	0.038	0.87	0.035	−0.020	0.029
3. Fort Cobb	0.77	0.052	−0.032	0.041	0.88	0.046	−0.021	0.041
4. Little River	0.73	0.074	0.065	0.035	0.79	0.064	0.054	0.035
5. Saint Joseph’s	0.46	0.073	0.052	0.051	0.60	0.079	0.055	0.057
6. South Fork	0.11	0.067	0.011	0.066	0.55	0.071	0.026	0.066
7. Reynolds Creek	0.74	0.050	−0.044	0.022	0.71	0.050	−0.044	0.022
8. Pali	—	—	—	—	—	—	—	—
9. Naqu	—	—	—	—	—	—	—	—
10. Yanco	0.71	0.067	0.044	0.051	0.59	0.061	0.038	0.047
11. Kyeamba	0.74	0.068	0.037	0.057	0.71	0.087	0.031	0.081
12. REMEDHUS	0.74	0.040	0.002	0.040	0.81	0.047	0.020	0.042
13. Benin	—	—	—	—	—	—	—	—
14. Niger	—	—	—	—	—	—	—	—
**Average**	**0.65**	**0.056**	**0.013**	**0.043**	**0.73**	**0.057**	**0.015**	**0.045**

**Table 6:** Statistical comparisons of NNsm, JAXAsm and LPRMsm with the *in situ* soil moisture for 2002–2019.

*In situ* SM	period	NNsm				AMSR_JAXA				AMSR_LPRM			
		CC	RMSE	Bias	ubRMSE	CC	RMSE	Bias	ubRMSE	CC	RMSE	Bias	ubRMSE
1. Walnut Gulch	2002–2019	0.51	0.043	0.024	0.035	0.35	0.054	−0.046	0.026	0.33	0.077	−0.041	0.065
2. Little Washita	2007–2019	0.61	0.044	0.003	0.044	0.41	0.106	−0.095	0.048	0.48	0.099	0.031	0.094
3. Fort Cobb	2007–2019	0.67	0.058	−0.037	0.045	0.48	0.122	−0.113	0.047	0.42	0.086	0.012	0.085
4. Little River	2002–2018	0.64	0.076	0.053	0.054	0.33	0.082	−0.032	0.076	0.29	0.257	0.200	0.161
5. Saint Joseph’s	2007–2019	0.41	0.069	0.042	0.055	0.17	0.123	−0.094	0.080	0.25	0.149	0.096	0.114
6. South Fork	2013–2018	0.42	0.047	0.007	0.046	0.35	0.121	−0.098	0.071	0.33	0.210	0.158	0.138
7. Reynolds Creek	2002–2018	0.48	0.059	−0.013	0.058	0.41	0.120	−0.098	0.061	0.59	0.130	0.076	0.105
8. Pali	2015–2016	0.43	0.076	−0.070	0.032	0.46	0.123	−0.120	0.028	0.44	0.139	0.125	0.061
9. Naqu	2010–2016	0.78	0.085	0.023	0.081	0.62	0.127	−0.058	0.113	0.82	0.092	0.076	0.052
10. Yanco	2006–2018	0.73	0.093	0.047	0.081	0.39	0.089	−0.052	0.072	0.53	0.121	0.088	0.082
11. Kyeamba	2006–2018	0.62	0.130	0.091	0.093	0.40	0.110	−0.074	0.082	0.37	0.146	0.093	0.112
12. REMEDHUS	2005–2018	0.82	0.038	0.011	0.036	0.57	0.081	−0.069	0.043	0.71	0.180	0.158	0.086
13. Benin	2006–2014	0.77	0.116	0.101	0.055	0.53	0.068	−0.037	0.057	0.48	0.302	0.219	0.208
14. Niger	2006–2014	0.46	0.029	0.006	0.029	0.65	0.082	0.064	0.053	0.60	0.067	0.051	0.043
**Average**		**0.60**	**0.069**	**0.021**	**0.053**	**0.44**	**0.101**	−0**.066**	**0.061**	**0.48**	**0.147**	**0.096**	**0.105**

### Validation and comparison with AMSR-E/AMSR2 standard products

**Fig. 7:**
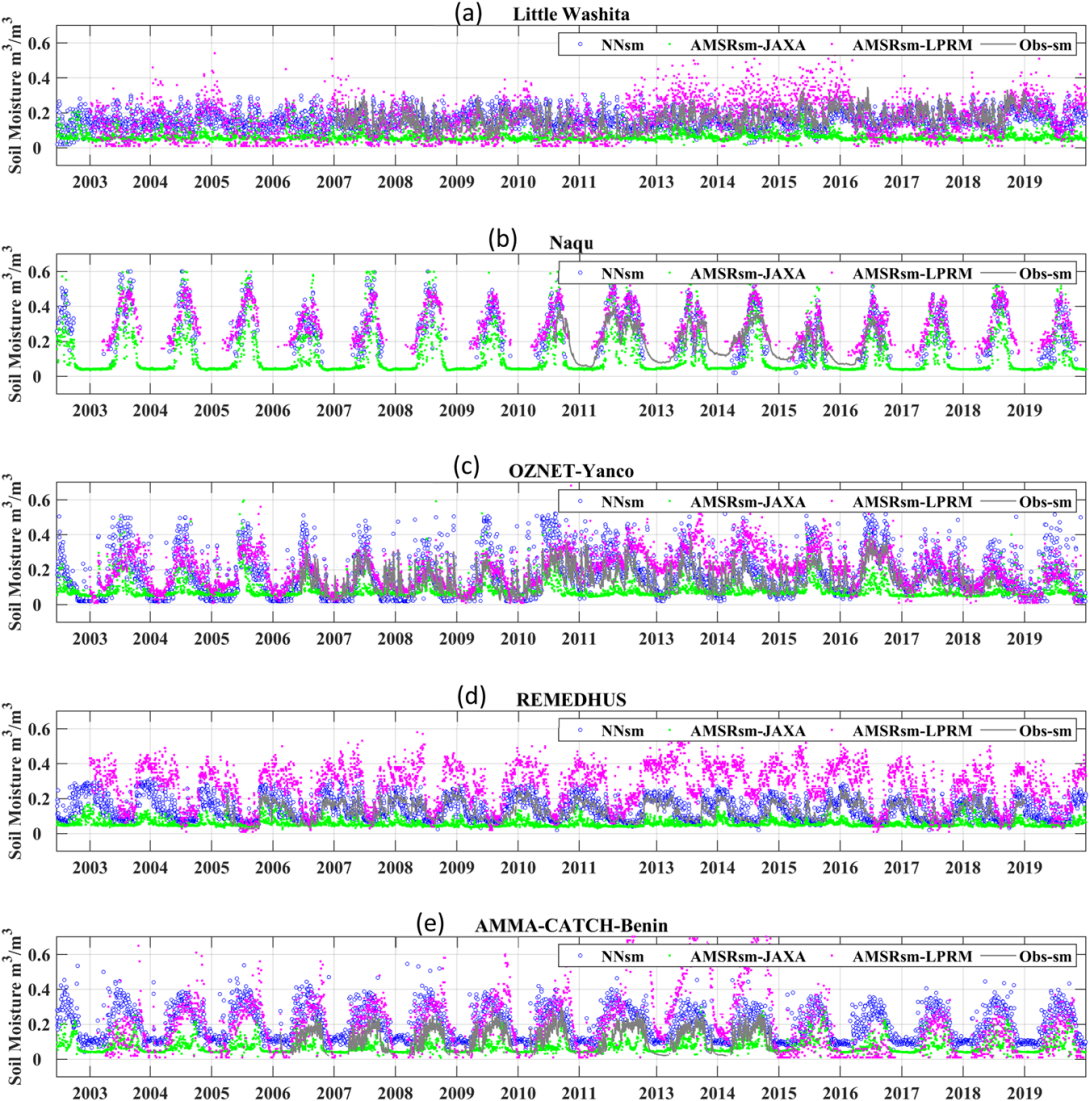
Time series of NNsm (blue dots) against AMSRsm-JAXA(green dots), the AMSRsm-LPRM (magenta dots), and the *in situ* soil moisture (Obs-sm in grey lines) for 2002–2019. *In situ* sites shown: (**a**) USDA-Little Washita, (**b**) Tibet-Naqu, (**c**) OZNET-Yanco, (**d**) REMEDHUS, and (**e**) AMMA-CATCH-Benin sites.

Moreover, to clarify the advantages of our algorithm and soil moisture products, we validated the performance of NNsm by comparing the simulated output with the satellite standard SSM products of AMSR-E/AMSR2 from JAXA and LPRM, JAXAsm and LPRMsm respectively, over the *in situ* sites. Results are shown in Fig. 7 and Table 6 (2002–2019). When calculating the statistical matrix, we use the intersection of the observational periods for all four datasets (NNsm, JAXAsm, LPRMsm, *in situ*), as shown in second column of Table 6. We also performed inter-comparisons separately over the AMSR-E period (2002–2011) and AMSR2 period (2012–2019) (see Supplementary Tables 1 and 2).

From the time series plots shown in Fig. 7 and the statistical comparison shown in Table 6, NNsm is generally consistent with *in situ* SSM, while NNsm may underestimate or overestimate soil moisture slightly at a few sites. The performance of NNsm is better than that of AMSR-E/AMSR2 SSM from JAXA and LPRM, with higher CC, lower RMSE and ubRMSE. In most sites, LPRM overestimated the soil moisture, while JAXA underestimated the soil moisture. LPRM in particular shows changes in bias and variability over time.

From the time series plots shown in Fig. 7 and the statistical comparison shown in Table 6, NNsm is generally consistent with *in situ* SSM, albeit with some scaling bias apparent in some sites (e.g., Fig. 7(e)). The JAXAsm and LPRMsm time series show biases as well, with JAXAsm typically underestimating the *in situ* observations and LPRM generally overestimating (Fig. 7(a–e), Table 6). The LPRMsm also displays some large changes in variability and mean state (Fig. 7(a,c,e)) which seem to not follow either the *in situ* observations or the main line of TB forcing that drive the JAXAsm and NNsm time series. Across the *in situ* validation sites, NNsm displays broadly lower biases, lower RMSEs and unbiased RMSEs (ubRMSE), and higher correlations with the *in situ* data than the JAXAsm and LPRMsm AMSR-E/2 soil moisture time series. This suggests that the NNsm may be providing added value from interactions between the reflectivities and microwave vegetation indices used as inputs in Eqs. 2–4 beyond what is used in the JAXA and LPRM retrieval algorithms.

### Comparison with SMOS and CCI soil moisture products

To quantify the utility of our algorithm and any benefits from creating an additional soil moisture product, we also compared NNsm with the satellite SMOS L3 SSM products(SMOSsm) (V300, https://www.catds.fr/) and CCI soil moisture product(CCIsm) (V05.2,combined)^35–37^, in terms of both accuracy and data volume.

CCIsm merges AMSR-E, Windsat and ASCAT from 2002 to 2010, and also merges SMOS and SMAP after 2010 and 2015 respectively; NNsm is derived from AMSR-E/AMSR2 TB from 2002 to 2019; SMOSsm is available from 2010. For appropriate comparison across these data spans, we carried out the comparison in two periods: one from 2002 to 2009, and the second from 2010 to 2019.

**Fig. 8:**
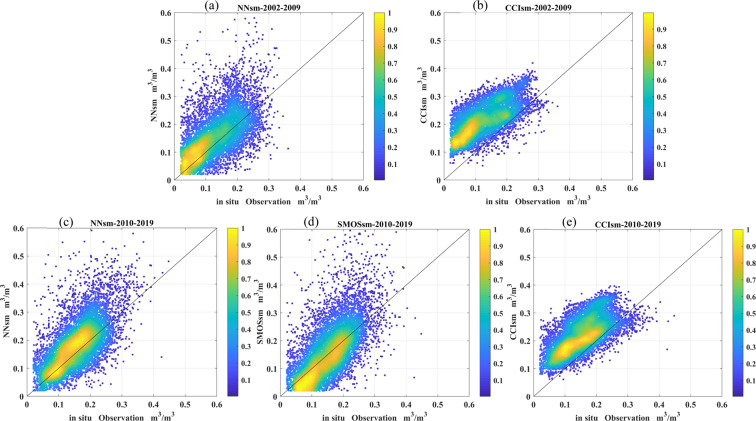
Jiont density scatter plot of *in situ* soil moisture (x-axis) against (**a**) NNsm and (**b**) CCIsm for 2010–2019, and (**c**) NNsm, (**d**) SMOSsm and (**e**) CCIsm for 2010–2019 over all 14 validation sites.

First, we quantify the relative accuracies of the SSM products. For the two periods (2002–2009 and 2010–2019) joint density scatter plots are shown in Fig. 8 and summary statistics in Tables 7–8, respectively. We also show data product intercomparison scatter plots for each site in Figs. S1–2 of the Supplementary file. Results are all shown for the overlapping data period; for example, data from the Little Washita validation site in Fig. 8 and Table 7 span the overlapping data windows of the NNsm data, the CCIsm data, and the *in situ* data from 2007 to 2009.

**Table 7:** Statistical comparisons of NNsm, and CCIsm with *in situ* soil moisture for 2002–2009.

*In situ* SM	period	NNsm				CCIsm			
		CC	RMSE	Bias	ubRMSE	CC	RMSE	Bias	ubRMSE
1. Walnut Gulch	2002–2009	0.62	0.048	0.021	0.043	0.67	0.110	0.106	0.032
2. Little Washita	2007–2009	0.61	0.050	0.019	0.046	0.57	0.074	0.057	0.046
3. Fort Cobb	2007–2009	0.64	0.075	−0.057	0.050	0.55	0.057	0.036	0.044
4. Little River	2002–2009	0.63	0.075	0.040	0.063	0.72	0.056	0.045	0.033
5. Saint Joseph’s	2007–2009	0.35	0.053	−0.002	0.053	0.69	0.118	0.113	0.034
6. South Fork^a^	—	—	—	—	—	—	—	—	—
7. Reynolds Creek	2002–2009	0.62	0.054	0.019	0.050	0.66	0.089	0.073	0.050
8. Pali^a^	—	—	—	—	—	—	—	—	—
9. Naqu^a^	—	—	—	—	—	—	—	—	—
10. Yanco	2006–2009	0.63	0.114	0.055	0.100	0.80	0.074	0.059	0.045
11. Kyeamba	2006–2009	0.47	0.135	0.086	0.104	0.71	0.090	0.071	0.056
12. REMEDHUS	2005–2009	0.81	0.042	0.017	0.039	0.85	0.118	0.114	0.031
13. Benin	2006–2009	0.76	0.131	0.116	0.061	0.77	0.135	0.127	0.045
14. Niger	2006–2009	0.21	0.033	−0.007	0.033	0.55	0.175	0.170	0.042
**Average**		**0.58**	**0.074**	**0.028**	**0.058**	**0.69**	**0.100**	**0.088**	**0.042**

^a^There is no *in situ* observations over those 3 sites for period (2002–2009).

**Table 8:** Statistical comparisons of NNsm,SMOSsm and CCIsm with *in situ* soil moisture for period of 2010–2019.

*In situ* SM	period	NNsm				SMOSsm				CCIsm			
		CC	RMSE	Bias	ubRMSE	CC	RMSE	Bias	ubRMSE	CC	RMSE	Bias	ubRMSE
1. Walnut Gulch	2010–2019	0.71	0.030	0.002	0.030	0.75	0.047	0.008	0.046	0.72	0.08	0.074	0.031
2. Little Washita	2010–2019	0.65	0.046	−0.001	0.046	0.71	0.057	−0.007	0.057	0.80	0.039	0.017	0.035
3. Fort Cobb	2010–2019	0.72	0.053	−0.031	0.044	0.76	0.065	−0.034	0.056	0.75	0.056	0.043	0.036
4. Little River	2010–2018	0.72	0.077	0.063	0.043	0.54	0.101	0.077	0.065	0.78	0.052	0.042	0.029
5. Saint Joseph’s	2010–2019	0.58	0.078	0.061	0.050	0.65	0.066	0.005	0.066	0.78	0.139	0.136	0.029
6. South Fork	2010–2018	0.49	0.047	0.010	0.046	0.64	0.072	0.007	0.071	0.65	0.089	0.083	0.033
7. Reynolds Creek	2010–2018	0.57	0.056	−0.016	0.054	0.59	0.074	−0.045	0.058	0.65	0.085	0.067	0.051
8. Pali	2015–2016	0.50	0.080	−0.076	0.027	0.10	0.132	0.021	0.131	0.37	0.096	0.092	0.027
9. Naqu	2010–2016	0.65	0.084	0.031	0.079	0.42	0.114	−0.009	0.114	0.79	0.039	−0.012	0.037
10. Yanco	2010–2018	0.74	0.093	0.051	0.077	0.80	0.071	0.036	0.062	0.82	0.060	0.041	0.044
11. Kyeamba	2010–2018	0.63	0.127	0.088	0.091	0.67	0.073	0.014	0.071	0.71	0.048	0.048	0.060
12. REMEDHUS	2010–2018	0.78	0.040	0.018	0.036	0.66	0.057	−0.02	0.054	0.76	0.101	0.101	0.032
13. Benin	2010–2014	0.82	0.103	0.091	0.048	0.68	0.123	0.091	0.083	0.88	0.113	0.113	0.036
14. Niger	2010–2014	0.40	0.036	0.003	0.036	0.60	0.070	0.032	0.062	0.49	0.143	0.143	0.047
**Average**		**0.64**	**0.068**	**0.021**	**0.051**	**0.61**	**0.080**	**0.013**	**0.071**	**0.71**	**0.084**	**0.071**	**0.038**

### 2002 to 2009: CCIsm, NNsm, and *in situ* data

In general, as evident in Fig. 8(a,b) and Table 7 the NNsm have substantially lower bias than CCIsm relative to the *in situ* observations, leading to a lower RMSE with roughly equivalent correlations. The ubRMSE is lower for the CCIsm, suggesting that the CCIsm bias is a primary source of error in the data set. CCIsm tends to overestimate the *in situ* values, especially when soil is dry (SSM < ~0.2 m^3^/m^3^); the average bias across sites is 0.088 m^3^/m^3^. Scatter plots of CCIsm vs *in situ* observations by site show some data processing artifacts as well (discretized SSM values evident in Supplementary Fig. 1.1, 1.7, and 1.12) which may introduce some errors as well.

### 2010 to 2019: CCIsm, NNsm, SMOSsm, and *in situ* data

In the L-bad era, as shown in Fig. 8(c,d,e) and Table 8, the three SSM products — NNsm, SMOSsm and CCIsm — have similar accuracies relative to the 14 *in situ* network sites. NNsm and SMOSsm have average biases of 0.021 m^3^/m^3^ and 0.013 m^3^/m^3^, and ubRMSEs of 0.051 m^3^/m^3^ and 0.071 m^3^/m^3^, respectively. Most NNsm and SMOSsm observations are located around the one-to-one line, while both products have some overestimated outliers. CCIsm has the lowest ubRMSE, with an average value of 0.038 m^3^/m^3^. But again CCIsm tends to overestimate the SM values at the *in situ* sites, particularly when soil is dry, with an average bias of 0.071 m^3^/m^3^.

**Fig. 9:**
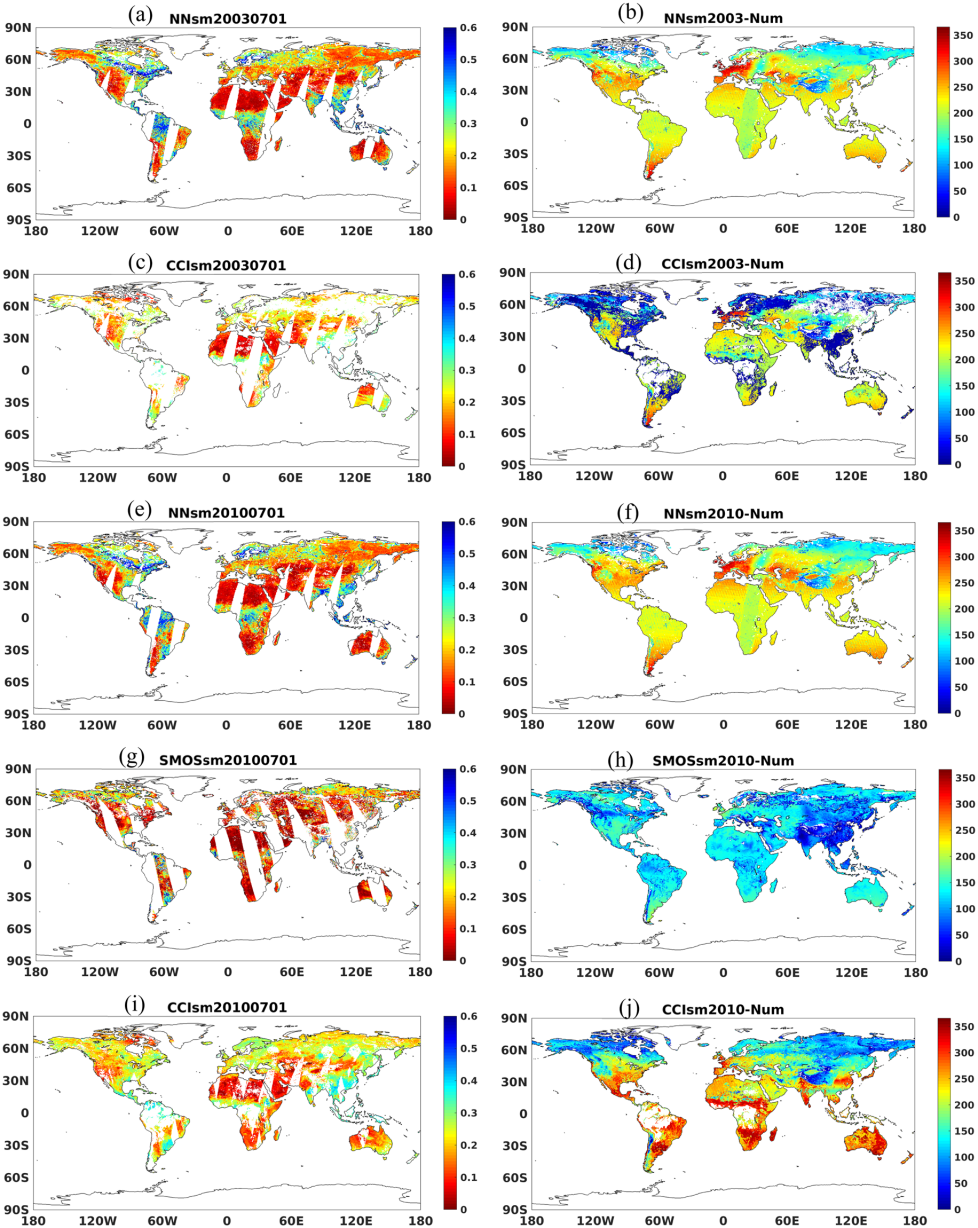
Global map of daily soil moisture(20030701) and the number of soil moisture retrievals over each grid in one year(2003): (**a**) NNsm, (**b**) number of NNsm observations per grid cell, (**c**) CCIsm, and (**d**) number of CCIsm observations per grid cell. Global map of daily soil moisture(20100701) and the number of soil moisture retrievals over each grid in one year(2010): (**e**) NNsm, (**f**) number of NNsm observations per grid cell, (**g**) SMOSsm, (**h**) number of SMOSsm observations per grid cell,(**i**) CCIsm, and (**j**) number of CCIsm observations per grid cell.

Secondly, the comparison is carried out in terms of the number of data. For the period from 2002 to 2009, we take 2003 as an example. As shown in Fig. 9(a), NNsm can provide global product in summer. In general, NNsm provides more than 200 soil moisture retrievals over each grid in middle latitudes, and provides more than 100 soil moisture retrievals over each grid in high latitudes, as shown in Fig. 9(b). CCIsm doesn’t have soil moisture retrievals over equatorial zone and most area of Russia (Fig. 9(c)). In the North America, Northern Europe, Southeast China, and the Tibetan Plateau, CCIsm has a few soil moisture retrievals, with a number less than 50 over each grid (Fig. 9(d)).

For the sake of application utility, we also compare data volumes for the three data sets. Example data volumes for the pre-L-band period (2002 to 2009), and L-band period (2010 to 2019) are shown in Fig. 9(a–d), Fig. 9(e–j), respectively. For the period from 2002 to 2009, we take 2003 as an example. As shown in Fig. 9, NNsm provides a global product in the boreal summer. In general, NNsm provides more than 200 soil moisture retrievals over each grid cell in the mid-lattitudes, and provides more than 100 soil moisture retrievals over each grid cell in the high lattitudes. CCIsm does not have soil moisture retrievals over much of the equatorial zone and most area of Russia. In North America, Northern Europe, southeastern China, and the Tibetan Plateau CCIsm has very few soil moisture retrievals, less than 50 per grid cell.

For period from 2010 to 2019, we take 2010 as an example. As shown in Fig. 9(e,g,i), the three products have a similar spatial pattern and dynamic range in summer. NNsm can provide considerable number of soil moisture retrievals globally except in Tibetan Plateau (Fig. 9(f)). SMOS has 150 soil moisture retrievals on the average but less retrievals in Asia, affected by RFI seriously (Fig. 9(h)). CCIsm has evident advantages in number of retrievals after 2010, but still has no retrievals over equatorial zone, and has less retrievals in most area of Russia, in North America, and the Tibetan Plateau (Fig. 9(j)).

For the 2010–2019 period, we highlight 2010 as an example. As shown in Fig. 9(e,g,i), the three products have similar spatial pattern and dynamic range in boreal summer. NNsm has an annual data volume of typically more than 200 retrievals per year outside of regions with permanent snow and ice cover. SMOS has 150 soil moisture retrievals per year on average, with fewer retrievals in Asia, significantly affected by RFI. CCIsm has particularly high data volumes in the subtropics, but fewer in cold regions (and masks retrievals for dense tropical vegetation).

### Potential benefits and usages of this dataset

#### Complement to SMAP product

The NNsm dataset can be seamlessly merged with SMAP SSM at daily scale, providing greater spatial coverage and higher frequency observations, as well as serving as a complementary gap-filling product for SMAP SSM (e.g., when SMAP entered safe mode temporarily from June 20 to July 22 in 2019).

**Fig. 10:**
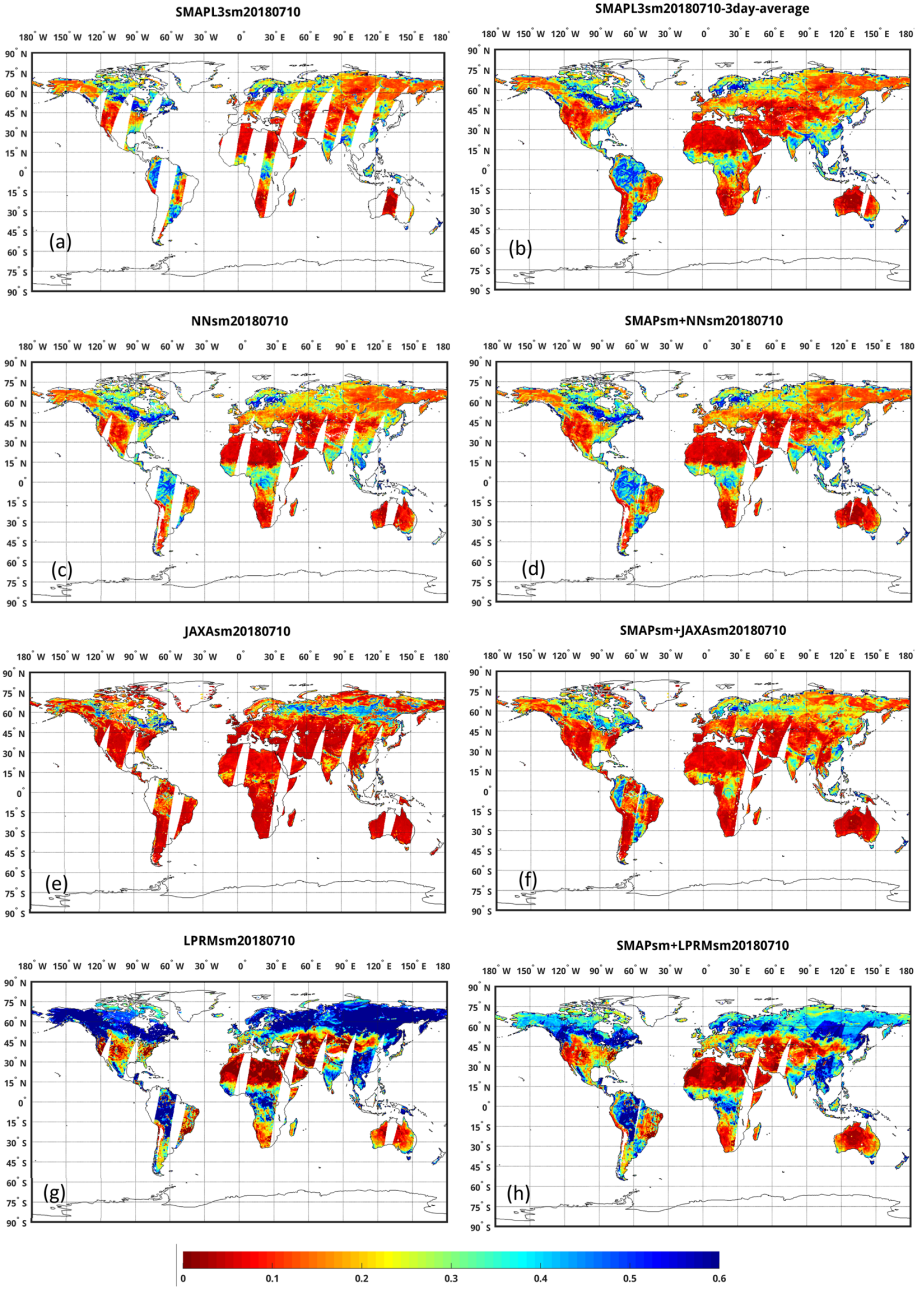
Global map of SMAPL3sm, NNsm, JAXAsm, LPRMsm and gap-filled SM products for July 10, 2018 (m^3^/m^3^). (**a**) SMAPL3sm, (**b**) 3-day average of SMAPL3sm, (**c**) NNsm, (**d**). SMAPL3sm gap-filled with NNsm, (**e**) JAXAsm, (**f**) SMAPL3sm gap-filled with JAXAsm, (**g**) LPRMsm, and (**h**) SMAPL3sm gap-filled with LPRMsm.

Figure 10 shows global maps of SMAPL3sm, NNsm, JAXAsm, LPRMsm, and a combined NNsm/SMAPL3sm. SMAP has less daily spatial coverage, and can have a global coverage with 3-day average, as shown in Fig. 10(a,b). NNsm derived from AMSR-E/2 has wider daily spatial coverage than SMAP (Fig. 10(c)). When combining NNsm with SMAPsm for the same day as shown in Fig. 10(d), for example, in China, Eastern and Southern Europe, the United States, South America, Africa and Australia, the combined map shows more coverage and provides almost full global coverage, with no obvious dataset discrepancies or inconsistencies. JAXAsm (Fig. 10(e)) has a drier soil moisture estimation and LPRMsm (Fig. 10(g)) has more wet soil moisture estimation at global scale. When merging them with SMAPsm separately, the fusion maps have obvious underestimation or overestimation, with noticeable striping in South America and East Asia (Fig. 10(f,h)).

**Fig. 11:**
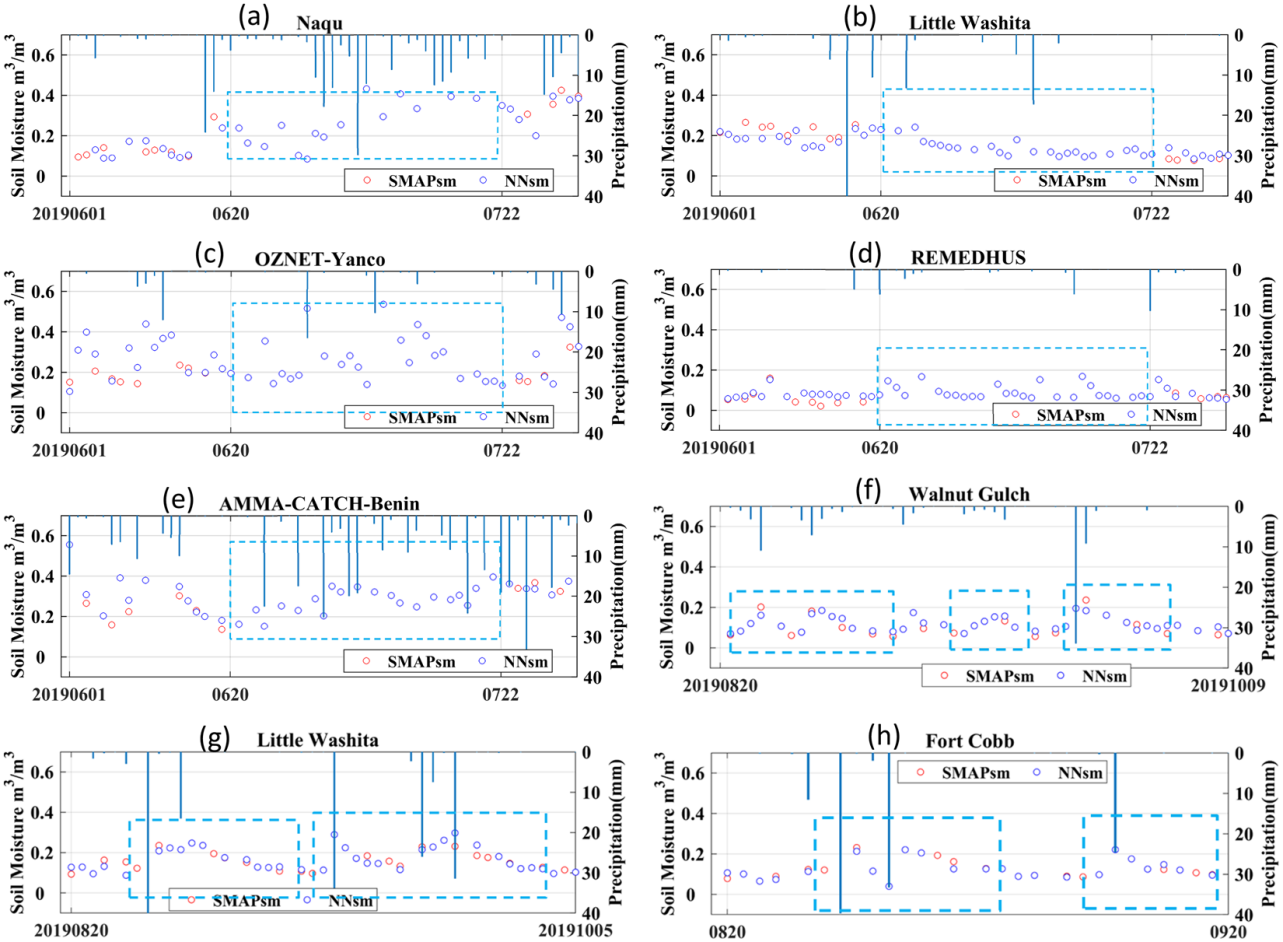
Time series of NNsm (blue dots), SMAPL3sm (red dots) and precipitation (blue bars) in 2019 when SMAP stopped capturing data temporarily (blue boxes). (**a**) USDA-Little Washita, (**b**) Tibet-Naqu, (**c**) OZNET-Yanco, (**d**) REMEDHUS and (**e**) AMMA-CATCH-Benin sites. Dry-down after rainfall.Time series of NNsm (blue dots), SMAPL3sm (red dots) and precipitation (blue bars) in 2019 over USDA sites (**a**) Walnut Gulch, (**b**) Little Washita, and (**c**) Fort Cobb.

SMAP was placed into safe mode and stopped capturing data for one month temporarily from June 20 to July 22 in 2019. During this period, SMAP provides no product, as marked in blue boxes shown in Fig. 11(a–e). NNsm is consistent and can capture the rainfall events with one-day delay, since the observation time of NNsm is 01:30 am. With a SMAP-similar accuracy, NNsm can provide complementary soil moisture for the SMAP SSM product.

#### Application for the study of short-term moisture dynamics

NNsm provides more frequent soil moisture observations for studying land-atmosphere interactions. SMAP has a narrow swath and can only provide roughly 10 measurements (or more dependent on latitude) within one month, while NNsm derived from AMSR-E/2 has a wider swath and provides measurements almost every day, as shown in Fig. 11(f–h). Combined with the standard SMAP product, NNsm can be used to extract dry-down curves with higher temporal resolution and process accuracy after rainfall.

#### Near-Real-Time product and extension to AMSR3

Having created and trained the models, it is now possible to produce near-real-time data products into the future provided that there are available brightness temperature data. Forward simulation of SM from TB data using the NNsm models is fast and efficient, and requires no ancillary datastreams. TB from future instruments can also be used, following a calibration to the existing AMSR2 data, analogous to the AMSRE-AMSR2 calibration performed in Eq. 1.

AMSR3 is scheduled to launch in 2023 as part of the GOSAT-GW mission and will provide similar C, X, and K band observations as a successor to AMSR2. Since our model only uses these observation bands as input, our method can be readily move to AMSR3 and a long term soil moisture product can continue to be generated for stable and consistent climatological studies of the terrestrial water cycle.

## Supplementary information

Supplementary File
