# Supplementary material for: A long term global daily soil moisture dataset derived from AMSR-E and AMSR2 (2002–2019)
Source: Sci Data. 2021 May 27;8:143. doi: 10.1038/s41597-021-00925-8 (PMC8160186; doi:10.1038/s41597-021-00925-8)
Supplement: Supplementary file 1 — Supplementary File [file 41597_2021_925_MOESM1_ESM.docx]

Supplementary

**Table S1.** Statistical comparisons of NNsm, JAXAsm and LPRMsm with the ground soil moisture for period (2002-2011).

| **In situ SM** | **period** | **NNsm** | | | | **AMSR_JAXA** | | | | **AMSR_LPRM** | | | |
| --- | --- | --- | --- | --- | --- | --- | --- | --- | --- | --- | --- | --- | --- |
|  |  | **CC** | **RMSE** | **Bias** | **ubRMSE** | **CC** | **RMSE** | **Bias** | **ubRMSE** | **CC** | **RMSE** | **Bias** | **ubRMSE** |
| 1.Walnut Gulch | 2002-2011 | 0.34 | 0.072 | 0.059 | 0.042 | 0.28 | 0.051 | -0.043 | 0.029 | 0.28 | 0.055 | -0.014 | 0.053 |
| 2.Little Washita | 2007-2011 | 0.55 | 0.052 | 0.027 | 0.044 | 0.53 | 0.088 | -0.078 | 0.04 | 0.56 | 0.07 | 0.008 | 0.07 |
| 3.Fort Cobb | 2007-2011 | 0.61 | 0.067 | -0.046 | 0.049 | 0.46 | 0.133 | -0.124 | 0.047 | 0.62 | 0.07 | -0.038 | 0.059 |
| 4.Little River | 2002-2011 | 0.59 | 0.079 | 0.041 | 0.067 | 0.33 | 0.084 | -0.036 | 0.076 | 0.64 | 0.135 | 0.115 | 0.071 |
| 5.Saint Joseph’s | 2007-2011 | 0.30 | 0.059 | 0.004 | 0.059 | 0.22 | 0.135 | -0.109 | 0.081 | 0.58 | 0.085 | 0.024 | 0.082 |
| 6.South Fork | - | - | - | - | - | - | - | - | - | - | - | - | - |
| 7.Reynolds Creek | 2002-2011 | 0.46 | 0.055 | 0.006 | 0.054 | 0.31 | 0.11 | -0.094 | 0.058 | 0.53 | 0.114 | 0.068 | 0.092 |
| 8.Pali | - | - | - | - | - | - | - | - | - | - | - | - | - |
| 9.Naqu | 2010-2011 | 0.82 | 0.090 | 0.042 | 0.079 | 0.65 | 0.135 | -0.084 | 0.106 | 0.86 | 0.086 | 0.069 | 0.051 |
| 10.Yanco | 2006-2011 | 0.64 | 0.122 | 0.067 | 0.103 | 0.29 | 0.093 | -0.042 | 0.083 | 0.61 | 0.091 | 0.06 | 0.069 |
| 11.Kyeamba | 2006-2011 | 0.56 | 0.145 | 0.104 | 0.101 | 0.37 | 0.119 | -0.077 | 0.091 | 0.56 | 0.132 | 0.099 | 0.087 |
| 12.REMEDHUS | 2005-2011 | 0.81 | 0.041 | 0.014 | 0.038 | 0.58 | 0.084 | -0.071 | 0.045 | 0.80 | 0.161 | 0.141 | 0.077 |
| 13.Benin | 2006-2011 | 0.70 | 0.134 | 0.119 | 0.062 | 0.47 | 0.069 | -0.04 | 0.056 | 0.55 | 0.156 | 0.125 | 0.093 |
| 14.Niger | 2006-2011 | 0.23 | 0.032 | -0.005 | 0.032 | 0.54 | 0.078 | 0.057 | 0.054 | 0.66 | 0.057 | 0.044 | 0.036 |
| **Average** |  | **0.55** | **0.079** | **0.036** | **0.061** | **0.42** | **0.098** | **-0.062** | **0.064** | **0.60** | **0.101** | **0.058** | **0.070** |

**Table S2.** Statistical comparisons of NNsm, JAXAsm and LPRMsm with the ground soil moisture for period (2012-2019).

| **In situ SM** | **period** | **NNsm** | | | | **AMSR_JAXA** | | | | **AMSR_LPRM** | | | |
| --- | --- | --- | --- | --- | --- | --- | --- | --- | --- | --- | --- | --- | --- |
|  |  | **CC** | **RMSE** | **Bias** | **ubRMSE** | **CC** | **RMSE** | **Bias** | **ubRMSE** | **CC** | **RMSE** | **Bias** | **ubRMSE** |
| 1.Walnut Gulch | 2012-2019 | 0.54 | 0.033 | 0.015 | 0.03 | 0.39 | 0.058 | -0.052 | 0.026 | 0.32 | 0.074 | -0.022 | 0.071 |
| 2.Little Washita | 2012-2019 | 0.74 | 0.039 | -0.014 | 0.037 | 0.39 | 0.118 | -0.107 | 0.048 | 0.33 | 0.111 | 0.051 | 0.098 |
| 3.Fort Cobb | 2012-2019 | 0.69 | 0.053 | -0.03 | 0.044 | 0.48 | 0.118 | -0.109 | 0.047 | 0.43 | 0.092 | 0.047 | 0.08 |
| 4.Little River | 2012-2018 | 0.74 | 0.077 | 0.067 | 0.038 | 0.32 | 0.081 | -0.027 | 0.076 | 0.19 | 0.356 | 0.308 | 0.178 |
| 5.Saint Joseph’s | 2012-2019 | 0.56 | 0.08 | 0.063 | 0.049 | 0.07 | 0.124 | -0.095 | 0.079 | 0.18 | 0.173 | 0.137 | 0.106 |
| 6.South Fork | 2013-2018 | 0.43 | 0.052 | 0.007 | 0.051 | 0.38 | 0.121 | -0.098 | 0.072 | 0.36 | 0.206 | 0.158 | 0.133 |
| 7.Reynolds Creek | 2012-2018 | 0.62 | 0.064 | -0.036 | 0.053 | 0.41 | 0.126 | -0.109 | 0.063 | 0.55 | 0.149 | 0.099 | 0.112 |
| 8.Pali | 2015-2016 | 0.56 | 0.08 | -0.073 | 0.033 | 0.52 | 0.122 | -0.118 | 0.029 | 0.57 | 0.133 | 0.114 | 0.069 |
| 9.Naqu | 2012-2016 | 0.76 | 0.084 | -0.006 | 0.084 | 0.63 | 0.128 | -0.074 | 0.105 | 0.81 | 0.082 | 0.06 | 0.056 |
| 10.Yanco | 2012-2018 | 0.76 | 0.082 | 0.044 | 0.069 | 0.50 | 0.089 | -0.062 | 0.064 | 0.49 | 0.14 | 0.11 | 0.086 |
| 11.Kyeamba | 2012-2018 | 0.63 | 0.121 | 0.086 | 0.085 | 0.45 | 0.108 | -0.078 | 0.075 | 0.16 | 0.158 | 0.095 | 0.127 |
| 12.REMEDHUS | 2012-2018 | 0.82 | 0.036 | 0.008 | 0.035 | 0.57 | 0.08 | -0.069 | 0.041 | 0.64 | 0.198 | 0.177 | 0.088 |
| 13.Benin | 2012-2014 | 0.87 | 0.09 | 0.081 | 0.04 | 0.64 | 0.065 | -0.035 | 0.055 | 0.68 | 0.452 | 0.384 | 0.238 |
| 14.Niger | 2012-2014 | 0.52 | 0.035 | 0.011 | 0.033 | 0.69 | 0.091 | 0.07 | 0.057 | 0.53 | 0.085 | 0.075 | 0.04 |
| **Average** |  | **0.66** | **0.066** | **0.016** | **0.049** | **0.46** | **0.102** | **-0.069** | **0.060** | **0.45** | **0.172** | **0.128** | **0.106** |


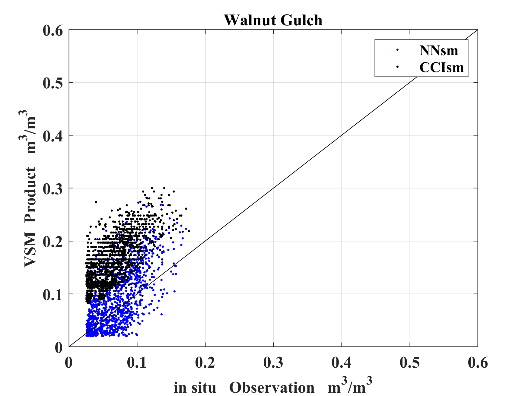


(1)


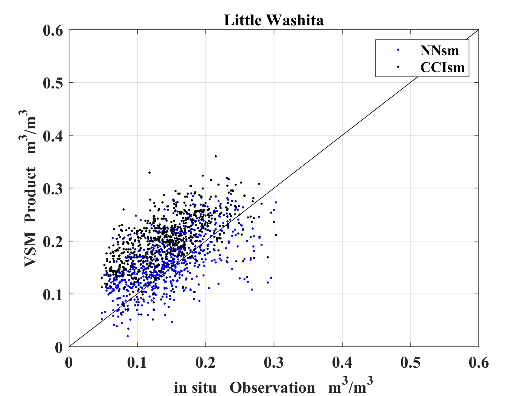


(2)


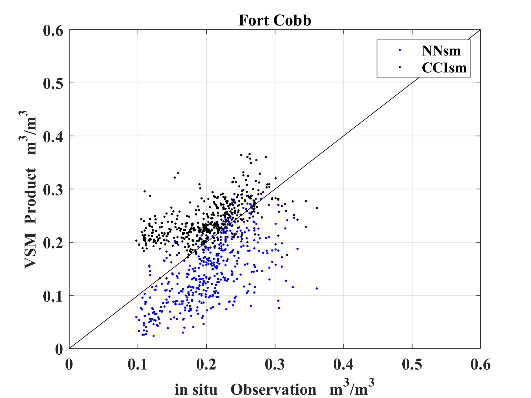


(3)


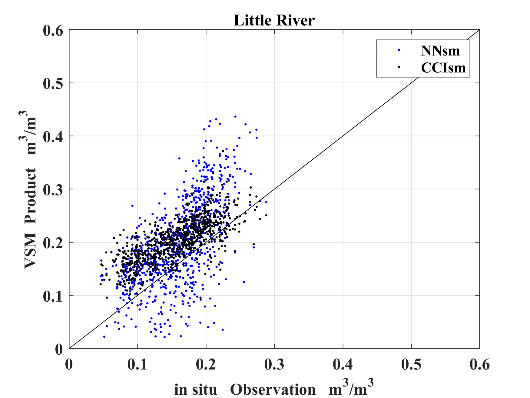


(4)


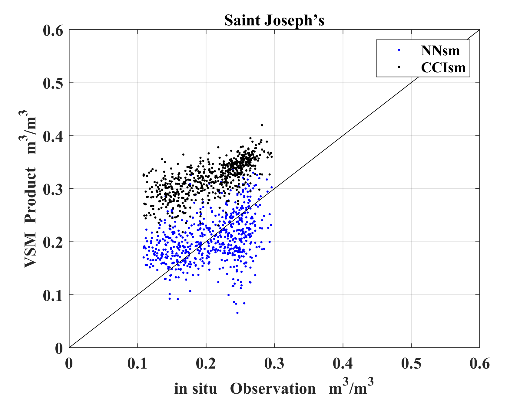


(5)


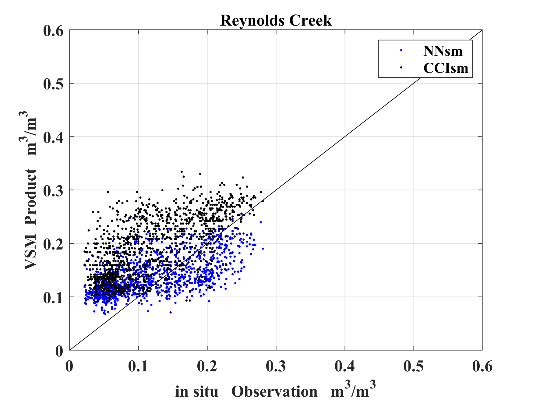


(7)


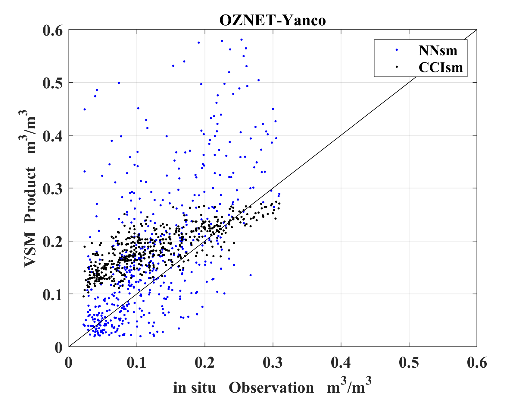


(10)


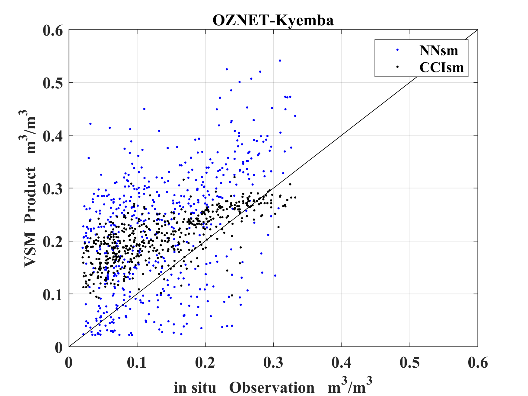


(11)


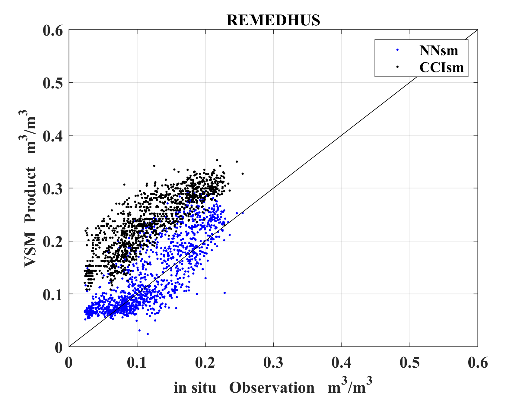


(12)


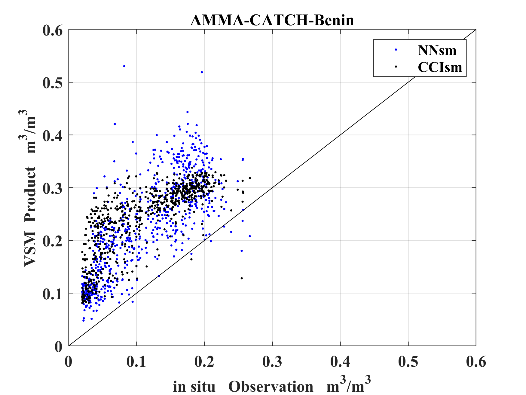


(13)


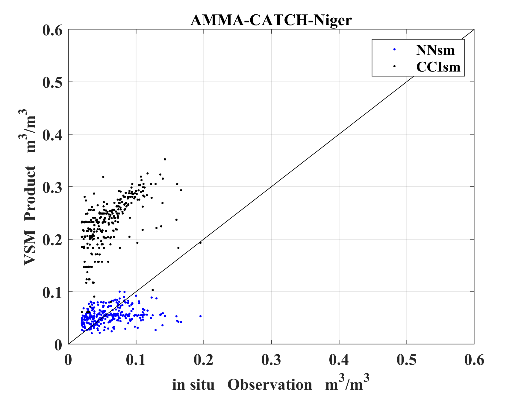


(14)

**Figure S1**. Scatter plot of in situ soil moisture (x-axis) against NNsm (blue dots), SMOSsm(red dots) and CCIsm(black dots) for 2002-2009 over sites: (1) Walnut Gulch, (2) Little Washita, (3) Fort Cobb, (4) Little River, (5) Saint Joseph’s, (7) Reynolds Creek, (10) Yanco, (11) Kyeamba, (12) REMEDHUS, (13) Benin, (14) Niger.


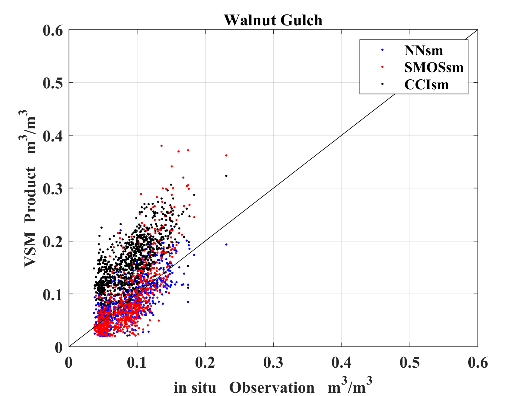


(1)


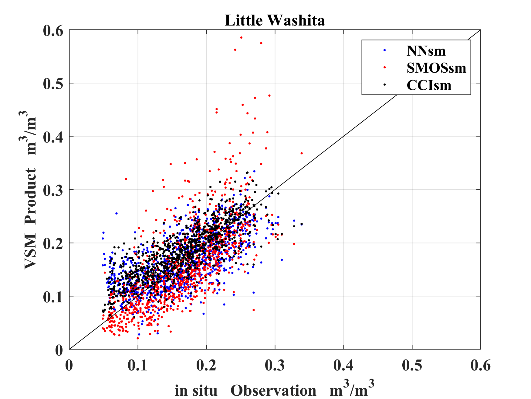


(2)


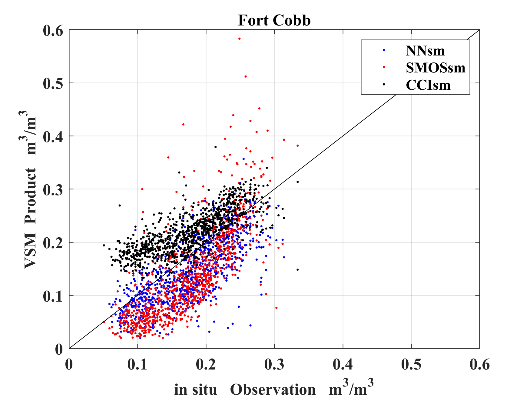


(3)


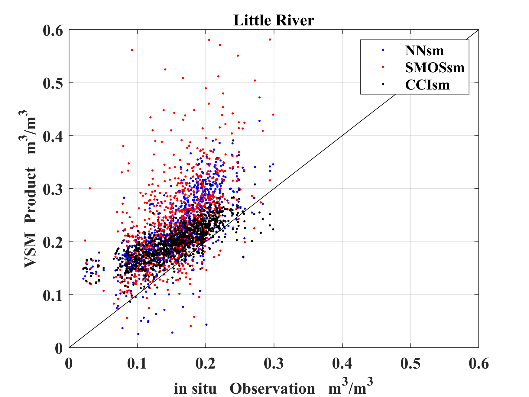


(4)


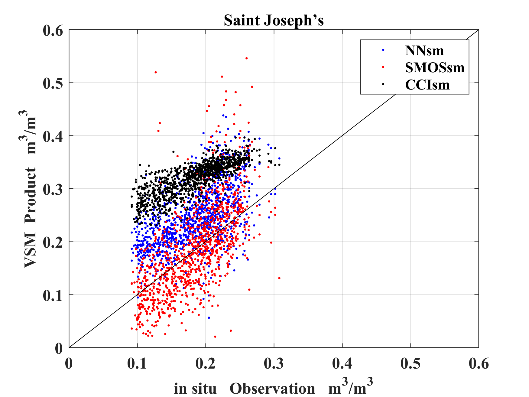


(5)


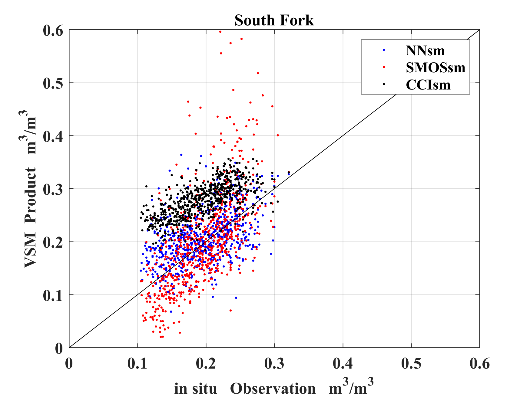


(6)


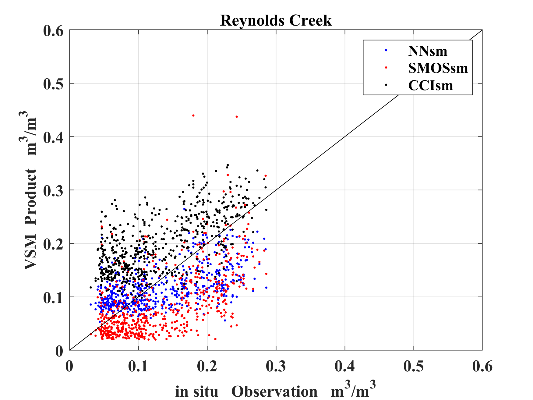


(7)


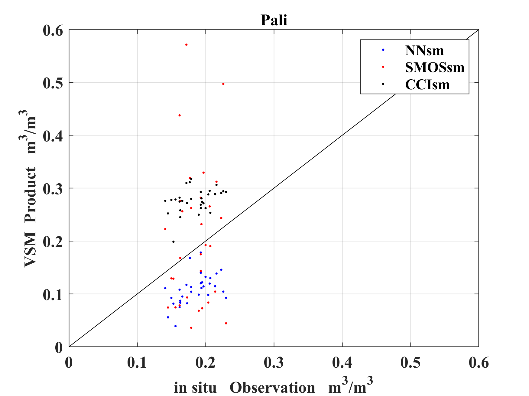


(8)


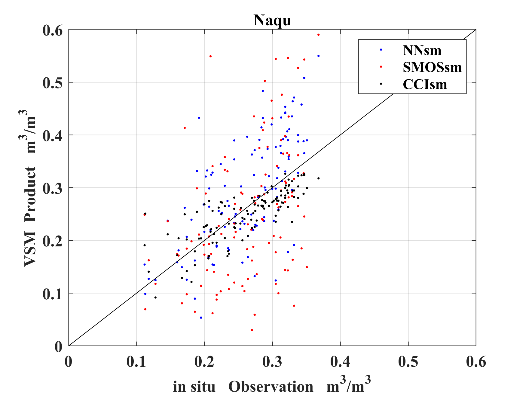


(9)


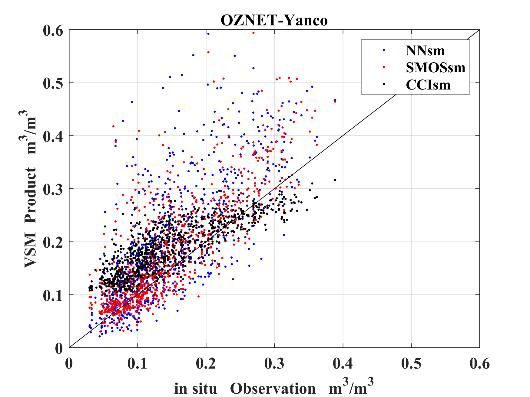


(10)


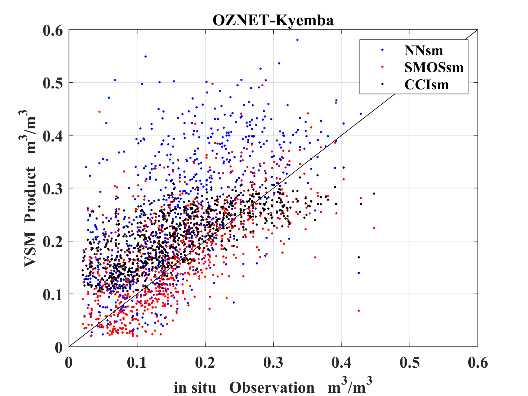


(11)


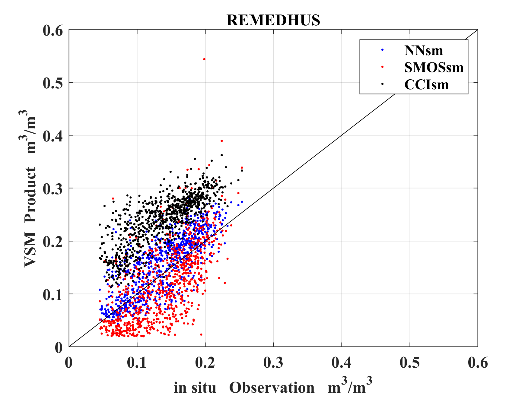


(12)


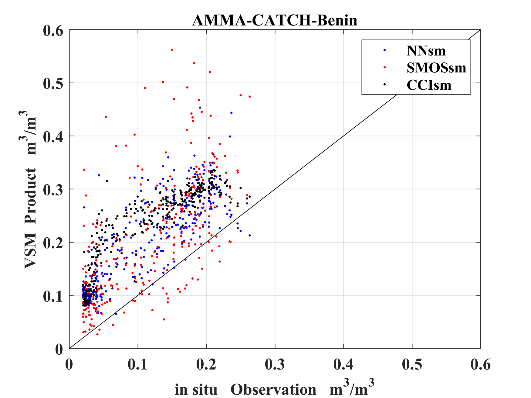


(13)


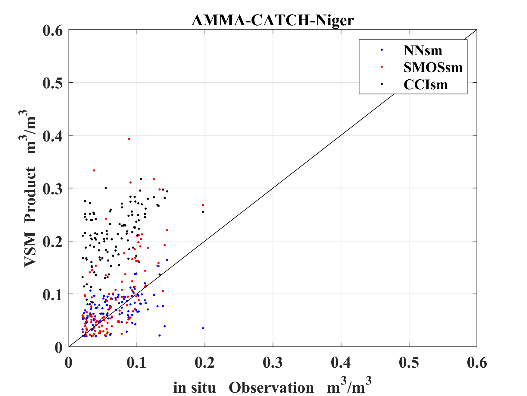


(14)

**Figure S2**. Scatter plot of in situ soil moisture (x-axis) against NNsm (blue dots),SMOSsm(red dots) and CCIsm(black dots) for 2010-2019 over sites: (1) Walnut Gulch, (2) Little Washita, (3) Fort Cobb, (4) Little River, (5) Saint Joseph’s, (6) South Fork, (7) Reynolds Creek, (8) Pali, (9) Naqu, (10) Yanco, (11) Kyeamba, (12) REMEDHUS, (13) Benin, (14) Niger.
